# 60-Hour Sleep Deprivation Affects Submaximal but Not Maximal Physical Performance

**DOI:** 10.3389/fphys.2018.01437

**Published:** 2018-10-16

**Authors:** Jani P. Vaara, Hermanni Oksanen, Heikki Kyröläinen, Mikko Virmavirta, Harri Koski, Taija Finni

**Affiliations:** ^1^Department of Leadership and Military Pedagogy, National Defence University, Helsinki, Finland; ^2^Faculty of Sport and Health Sciences, Neuromuscular Research Center, University of Jyväskylä, Jyväskylä, Finland; ^3^J7/Training Division, Finnish Defence Forces, Defence Command, Helsinki, Finland

**Keywords:** sleep loss, sleep deprivation, cardiorespiratory fitness, strength, motor control, balance

## Abstract

The effect of 60-h sleep deprivation (SD) on physical performance and motor control was studied. Twenty cadets were measured for aerobic performance (VO_2_) before and immediately after the SD period. Maximal strength and EMG of the knee extensor muscles were measured before and after 60 h of SD. Balance, reaction times and motor control were assessed every evening and morning during the SD period. Main effects were observed for heart rate (*p* = 0.002, partial eta squared: 0.669), VO_2_ (*p* = 0.004, partial eta squared: 0.621), ventilation (*p* = 0.016, partial eta squared: 0.049), and lactate concentration (*p* = 0.022, partial eta squared: 0.501), whereas RER remained unaltered (*p* = 0.213, partial eta squared: 0.166). Pairwise comparisons revealed decreased values at submaximal loads in heart rate, VO_2_, ventilation (all *p* < 0.05) but not in RER, whereas all of their respective maximal values remained unchanged. Moreover, pairwise comparisons revealed decreased lactate concentration at maximal performance but only at 8-min time point during submaximal workloads (*p* < 0.05). Pairwise comparisons of maximal strength, EMG and rate of force development revealed no change after SD. Main effects were observed for motor and postural control, as well as for reaction times (all *p* < 0.05), whereas pairwise comparison did not reveal a consistent pattern of change. In conclusion, motor control can mostly be maintained during 60-h SD, and maximal neuromuscular and aerobic performances are unaffected. However, submaximal cardiorespiratory responses seem to be attenuated after SD.

## Introduction

Although the primary function of sleep has only been poorly evidenced, sleep is being considered as a vital factor in daily life, affecting both mental and physiological functioning (Frank and Benington, 2006). Therefore, working ability can be compromised in occupations that include sleep restriction or total sleep deprivation. A condition involving a continuous time period overnight(s) without any sleep is called sleep deprivation (SD). SD is a factor affecting performance capacity in prolonged competitions in sports, such as e.g., ultramarathon races (Vernillo et al., 2016). However, it is not a common feature in any occupation but can occasionally occur in firefighter and military occupations as sustained operations, as well as in some instances in healthcare and industrial manufacturing (Caruso, 2014). In such security and safety occupations the working ability, including mental and physical capabilities, has to be maintained in spite of stressors such as SD. Physical capabilities are essential parts of working ability partly by their positive effect to resist and endure mental, physical and external stress sources. Therefore, physical fitness standards exist in the military and fire services including the key elements of physical fitness, such as aerobic performance, muscular strength, mobility and coordination (Kenny et al., 2016). Thus, it is of occupational relevance to study how acute SD affects physical performance.

Collectively, the previous studies, assessing the effect of SD on aerobic performance, have shown a tendency to a decreased time to exhaustion in most (Martin, 1981; Martin and Chen, 1984; Chen, 1991; Azboy and Kaygisiz, 2009; Temesi et al., 2013) but not all studies (Goodman et al., 1989; Racinais et al., 2004). Moreover, indices of aerobic performance such as heart rate and respiratory parameters have shown more controversial findings both at submaximal and maximal levels (Martin, 1981; Martin and Gaddis, 1981; Martin and Chen, 1984; Bond et al., 1986; Plyley et al., 1987; Chen, 1991). A recent review concluded that although the results remain somewhat mixed, there is evidence suggesting that SD may induce detrimental effects on physical performance (Fullagar et al., 2015). Partly, the controversial findings may relate to differences in methodological issues and study designs, such as the length of SD period and the fitness test used as well as an insufficient statistical power due to small sample sizes (Fullagar et al., 2015).

Whereas, there is some evidence indicating detrimental effects of SD on aerobic performance, the literature of the effects of SD on muscular strength and motor control is more limited. Most of the previous studies report muscular strength unchanged after SD (Symons et al., 1988b; Rodgers et al., 1995; Meney et al., 1998; Temesi et al., 2013), however, this is not a universal finding (Takeuchi et al., 1985). Regarding motor control and balance, most studies indicate a deleterious effect of SD (Fabbri et al., 2006; Forsman et al., 2007; Morad et al., 2007; Gomez et al., 2008; Bougard et al., 2011). Nevertheless, Patel et al. (2008) found that postural control decreased after 24 h of SD, however, the decrement did not continue after 36 h and was even partly recovered. Similarly, simple reaction times have mostly been observed to increase after SD (see e.g., Fullagar et al., 2015) however, no change has also been observed (Symons et al., 1988b).

The purpose of the present study was to investigate effects of 60-h SD on aerobic and neuromuscular performance, motor control and balance in young healthy participants. The rationale for the duration of SD in the present study comes from practical point of view: in military continuous operational work including SD can reach up to 48–60 h or even more (e.g., Nindl et al., 2002). It was hypothesized that 60-h SD would induce changes in cardiorespiratory performance but not in neuromuscular performance. Moreover, it was expected that decrements in motor control and reaction time would occur.

## Materials and methods

### Participants and study procedures

A convenience study sample consisted of twenty male cadets (age 26 ± 2 years, body height 1.77 ± 0.01 m, body mass 79.6 ± 11.1.kg) who volunteered for the study during their military training period. During the study period, the participants took part in their military training, which contained planning, designing and tracking defense operation in tandem with safe and security authorities. The military training period consisted of sedentary desk-based office work, as a military staff exercise. During the study period the participants were mainly sedentary and did not involve in other than occasional light physical activity (walking). When not on duty, the participants spend their time with sedentary activities, such as for example playing cards or reading. This study was carried out in accordance with the recommendations of University of Jyväskylä with written informed consent from all subjects. All subjects gave written informed consent in accordance with the Declaration of Helsinki. The protocol was approved by the ethical committee of the University of Jyväskylä (October 28, 2005).

**Figure 1:**
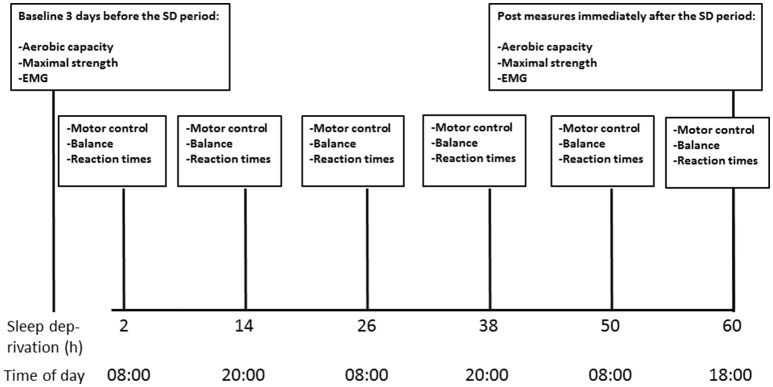
Timeline of the measurements during the 60-h SD period.

All subjects were healthy (free from chronic diseases) and physically active, performing moderate intensity exercise 3–5 times per week with a typical training session lasting 60–90 min. There were four smokers in the study subjects. Based on a circadian rhythm questionnaire, the subjects were classified as morning types and their daily rhythm of life was regular (Folkard et al., 1979). The subjects kept sleep logs for four nights preceding the study (mean sleep time 6:52 ± 2:28 h/night). They were tested for maximal aerobic performance, maximal strength, and electromyography (EMG) of the knee extensor muscles 3 days before (PRE) and immediately after (POST) the SD period. In addition, measures of motor control, balance and reaction times were measured every evening and morning (altogether six times) during the 60 h of SD. The tests were always started at the same time (8:00 p.m. and 8:00 a.m.). Familiarization to the testing protocol was done a day prior to the first measurements. Due to scheduling issues only 10 participants were randomly selected for the aerobic performance test (for the last 3 workloads: the number of subjects were 9, 8, and 4, respectively, whereas all other tests were completed by the whole study sample (*n* = 20). The baseline aerobic performance test was conducted 3 days before the beginning of the SD period. The overview on measurements during the study is shown in the Figure 1.

### Aerobic performance

Aerobic performance was measured with bicycle ergometer test (Ergoline, Ergometrics, 800s, Germany) using a ramp-protocol (Porszasz et al., 2003). The progressive test included 1-min loads starting from 100 W with 25 W increments each minute until exhaustion. The participants were ordered not to consume any food or caffeine containing drinks before the test. The subjects were ordered to keep the pedaling cadence within 60–90 rpm during the test with loud verbal encouragement in order to achieve the best possible result. The test was terminated when the cadence dropped below 60 rpm (for longer than 5 s). Thereafter, a 3-min active recovery period with 100 W was completed with individually chosen cadence. During the test, blood lactate was measured with a hand-held portable analyzer (LactaPro, Arkray, Inc., Tokyo, Japan) with 2-min intervals. A small (5 μl) blood sample was drawn from fingertip with the site standardized for a given subject. Blood lactate was also measured after the 3-min recovery period. The measurement of blood lactate is indicative of changes in anaerobic glycolysis during exercise and maximal performance. Heart rate (Polar Vantage, NV, Kempele, Finland) and respiratory gases (oxygen consumption (VO_2_), respiratory exchange ratio (RER), and ventilation (VE) were continuously measured (K4b2, Cosmed, Italy) and the rate of perceived exertion (RPE) was asked every 2 min.

### Neuromuscular performance

Maximal muscle strength and maximal rate of force development (RFD) of the knee extensors and patellar tendon tap reflexes were measured PRE and POST SD using a specifically constructed chair with a strain gauge for measuring force (Walker et al., 2009) and a pendulum for inducing a patellar tendon tap (University of Jyväskylä, Finland). Simultaneously, EMG was recorded from the vastus lateralis muscle with bipolar surface electrodes (Blue Sensor M with interelectrode distance of 2 cm, Ambu A/S, Ballerup, Denmark) that were placed longitudinally along presumed fascicle orientations over the muscle bellies between the center of the innervation zone and the distal tendon of each muscle. EMG data was collected via EISA (bandwidth 10 Hz to 1 kHz per 3 dB), a 10-channel EMG detection system (model: 16–2) (University of Freiburg, Germany) at a sampling frequency of 1,000 Hz. The signal was pre-amplified with a factor of 200 by an integrated preamplifier in the shielded cables. Analog-to-digital conversion of EMG and force data was done simultaneously through a Power1401 high-performance multi-channel data acquisition interface (Cambridge Electronic Design Ltd., Cambridge, England). Compatible Signal 4.0 (Cambridge Electronic Design Ltd., Cambridge, England) computer software was used for data recording, data reduction, and subsequent analyses. EMG data was high-pass filtered (2^nd^ order Butterworth filter, 12 dB/octave) with a cut-off frequency of 10 Hz.

Maximal isometric knee extension force was measured at knee joint angle of 107°. The performance with highest peak force of two to three attempts was further analyzed. RFD was assessed as the greatest slope using a 10 ms window. From the tendon tap, the peak-to-peak mechanical and EMG responses were analyzed (Avela et al., 2004).

### Motor control, balance, and reaction time

Target movement of the right hand was used to assess changes in motor control. The target movement was measured with a specific apparatus allowing horizontal movement of the forearm that was measured with a potentiometer (Bottas et al., 2005). Participants were sitting with their right shoulder joint abducted around 80°, forearm fixed in a semi-supinated position to the lever arm of the apparatus and the wrist stiffened by a spatula. The participants extended their elbow from the starting point to the end point with maximal speed and accuracy. The instructions for the participants were as follows: “perform always accurate and after a successful movement you may speed up the performance without sacrificing accuracy” (Kempf et al., 2001). They were given online visual feedback of the movement from a monitor. The experiments were conducted with 20° and 60° ranges of motion from 140° to 80° and from 120 to 100° (180° being full elbow extension). Five trials were done per each range of motion and the five results were averaged in both conditions. The variables of the target movement were error of the displacement (degrees) and peak angular velocity (deg·s^−1^) as a mean from a 20 ms time window.

*Balance* was measured with an equilateral triangle (800 mm) force platform system (Good Balance system, Metitur Ltd., Jyväskylä, Finland). A participant was positioned standing on the force platform where the system measures the vertical reaction forces, which are used for calculating the medio-lateral (x) and antero-posterior (y) coordinates of the displacement of the center of pressure from the base of support. The variables used in the present study were sagittal and frontal direction of swaying distance in millimeters. Postural sway velocity moment is defined as the average horizontal area covered by movement of the center (antero-posterior and medio-lateral direction) of force per second. The calculated error in the x and y coordinates of the center of pressure is <1.0 mm when the mass of the subject is at least 40 kg (Good Balance-User's manual 2000). During measurements, analog signals from the transducers were amplified for A/D conversion and recorded to a computer at a frequency of 50 Hz (Ha et al., 2014).

Four different balance tests were assessed on the force platform system: feet side-by-side Eyes Open (side-by-side EO) and Eyes Closed (side-by-side EC) and feet in tandem with eyes open (tandem EO) and closed (tandem EC) (e.g., Fabbri et al., 2006). In the tandem, the feet were positioned heel-to-toe along the midline of the platform. During the tandem EO tests, the participants were instructed to keep their arms down by their sides and gaze fixed at the marked point at eye level, two meters in the front of the participant. The outcomes of the balance test were there of medio-lateral distance (mm), antero-posterior distance (mm), and moment of velocity (mm/s) in all of these four positions.

*Reaction times* were measured in a quiet room using a simple reaction time test for light and sound stimulus with a custom device built in University of Jyväskylä. The participants sat in a chair having the device on a table in front of them. Initially, the participants held their dominant hand index finger on a starting button with the instruction to react as fast as possible to press another button (15 cm from the start button) when they saw a light blinking on top of the device. The same procedure was done with a sound stimulus. The device recorded the time (ms) when finger was released from the starting button and pressed the other button. The participants had three trials per each mode starting with light and followed by the sound mode of the reaction time test. Previously, sufficient reliability for reaction time assessment has been reported using similar procedures (Hamsher and Benton, 1977).

### Statistical analysis

A priori power analysis for PRE vs. POST difference for bicycle ergometer test for heart rate and VO2 variables (with a power = 0.80, an alpha = 0.05, and f = 0.4 for large effect size) suggested a sample size of *n* = 15. However, the measures had to be performed in a very tight schedule for all subjects to begin and finish the SD period at the same time. Therefore, only 10 subjects could be measured for aerobic fitness.

Standard statistical methods were used for the calculation of means, standard deviations and 95 % confidence intervals (CI). Normality and absence of large outliers were verified by using Shapiro-Wilks test, observing the normality plots, and residual plots. Repeated measures ANOVA (time × pre vs. post) with Bonferroni as a *post-hoc* test was used to test the effect of SD on aerobic performance variables, motor control, balance and reaction times. Sphericity corrections in repeated measures testing were utilized if Mauchly‘s test showed deviation from sphericity. *Post-hoc* pairwise comparisons for time effect were performed only if *F*-test *p*-value was significant. Additional pairwise *t*-tests were conducted for timepoints with <10 subjects at the end of the Vo2max test. All statistical analyses were done by using IBM Statistics software (SPSS 24.0.0.0).

## Results

### Aerobic performance

#### Maximal performance

After SD, there was no difference (*p* > 0.05) in time to exhaustion (PRE: 9:27 ± 1:22, POST: 10:06 ± 1:32 min:s), maximal heart rate (PRE: 190 ± 7, POST: 187 ± 6 bpm), maximal VO_2_ (PRE: 38.6 ± 8.1, POST: 38.5 ± 6.4 ml·kg^−1^·min^−1^), maximal ventilation (PRE: 149.6 ± 33.4, POST: 153.1 ± 30.2 L/min), maximal RER (PRE: 1.42 ± 0.30, POST: 1.30 ± 0.12), or rate of perceived exertion (PRE: 14 ± 1, POST: 14 ± 1). However, maximal blood lactate level was lower after SD (PRE: 10.7 ± 2.2; POST: 8.2 ± 2.4 mmol/L) (*p* < 0.05).

#### Submaximal performance

**Figure 2:**
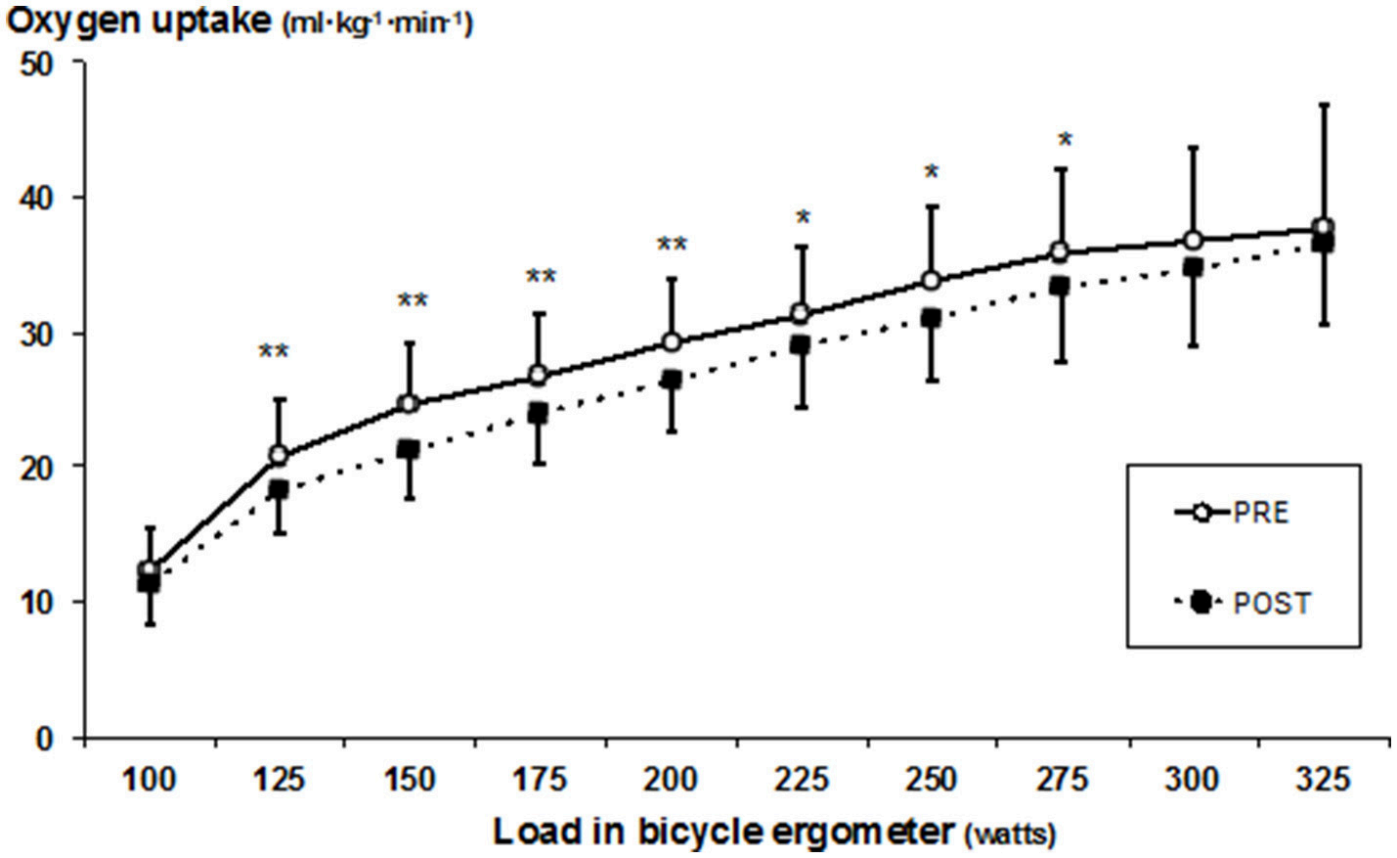
Mean (±SD) oxygen uptake during bicycle ergometer measured before (PRE) and after (POST) the SD test. Significant difference between PRE and POST values, *p < 0.05; **p < 0.005.

**Figure 3:**
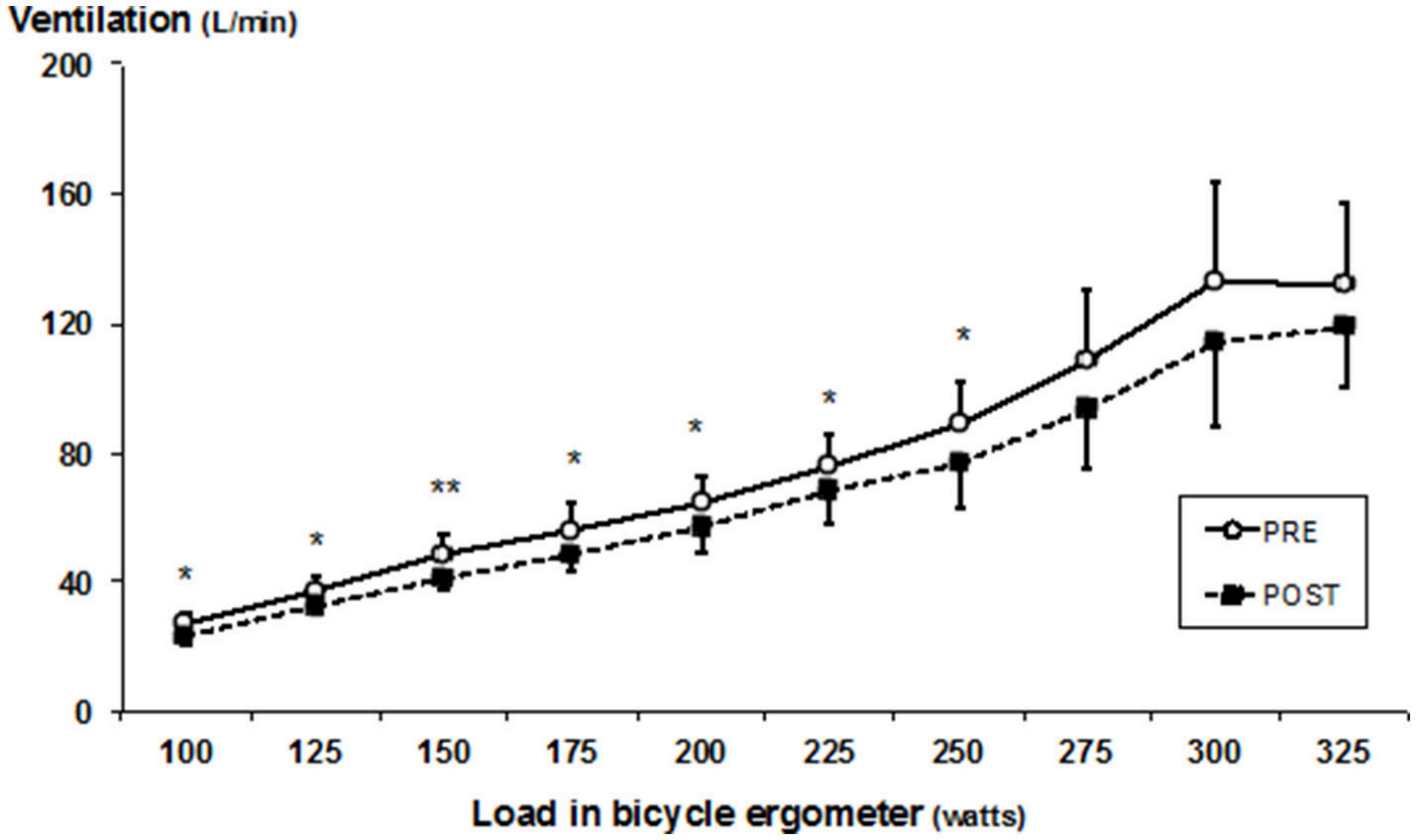
Mean (±SD) ventilation during bicycle ergometer test measured before (PRE) and after (POST) the SD. Significant difference between PRE and POST values, *p < 0.05; **p < 0.005.

**Figure 4:**
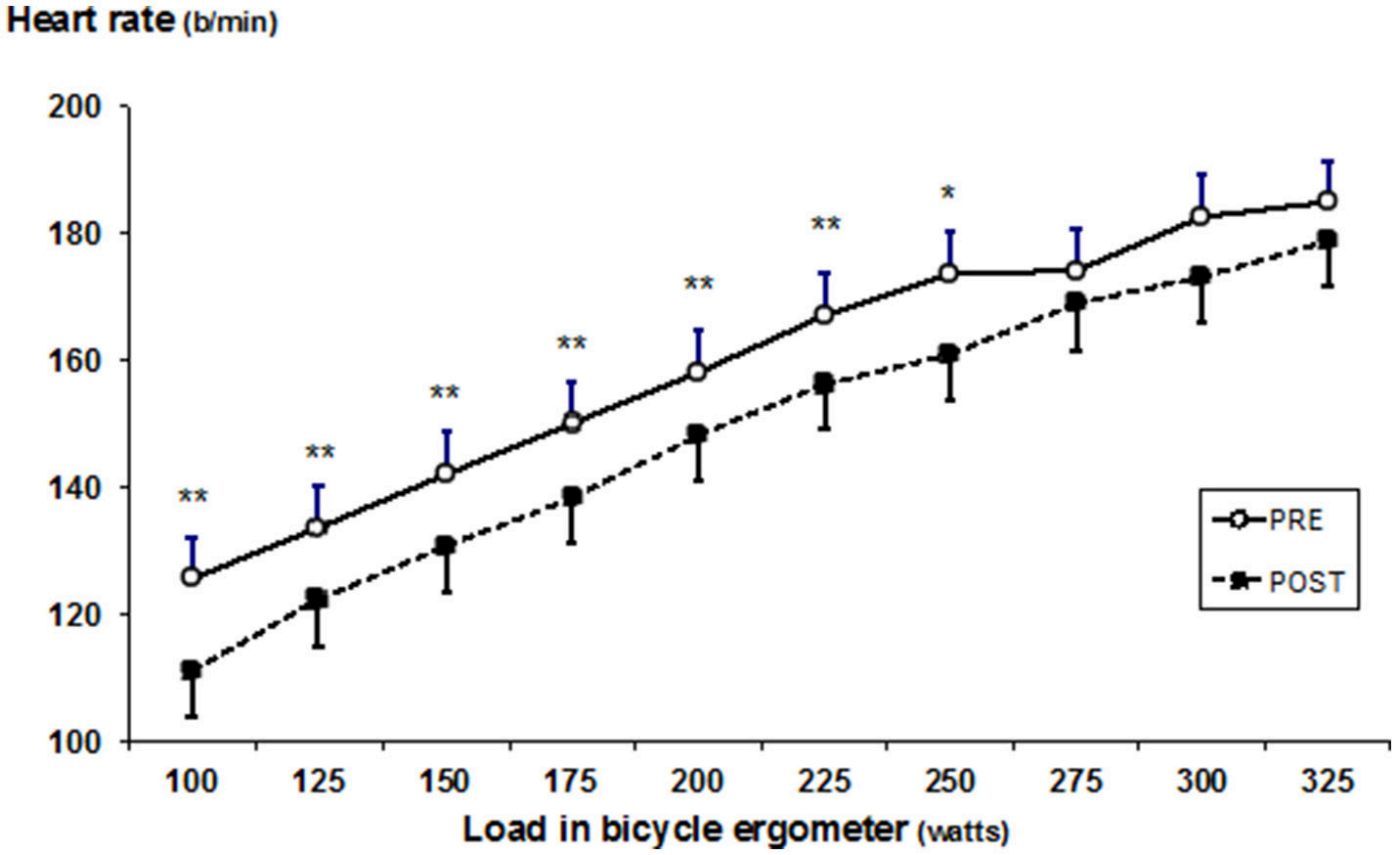
Mean (±SD) heart rate during bicycle ergometer test before (PRE) and after (POST) the SD. Significant difference between PRE and POST values, *p* < 0.05; **p < 0.005.

Heart rate, VO_2_, and ventilation decreased (all *p* < 0.05) in submaximal loads (100–250 W) (Figures 2–4), whereas RER (Supplementary Table 1) and RPE was unchanged after SD. In addition, a decrease was observed in mean heart rate (PRE: 160 ± 11, POST: 149 ± 9 bpm, *p* < 0.05), and mean VO_2_ (PRE: 28.6 ± 5.3, POST: 26.9 ± 4.1 ml·kg^−1^·min^−1^, *p* < 0.05), whereas mean ventilation (PRE: 73.5 ± 11.5, POST: 69.2 ± 10.5 l/min) and RER (PRE: 1.13 ± 0.12, POST: 1.08 ± 0.06) remained unchanged. Blood lactate decreased at a load of 275 W (at 8 min) (PRE: 9.4 ± 3.2; POST: 6.5 ± 1.6 mmol/L) (*p* < 0.05). The detailed performance test results including PRE-POST differences are shown in the Supplementary Table 1 and the statistical details including degree of freedom, mean square and effects size (partial eta square) in the Supplementary Table 2.

### Neuromuscular performance

**Figure 5:**
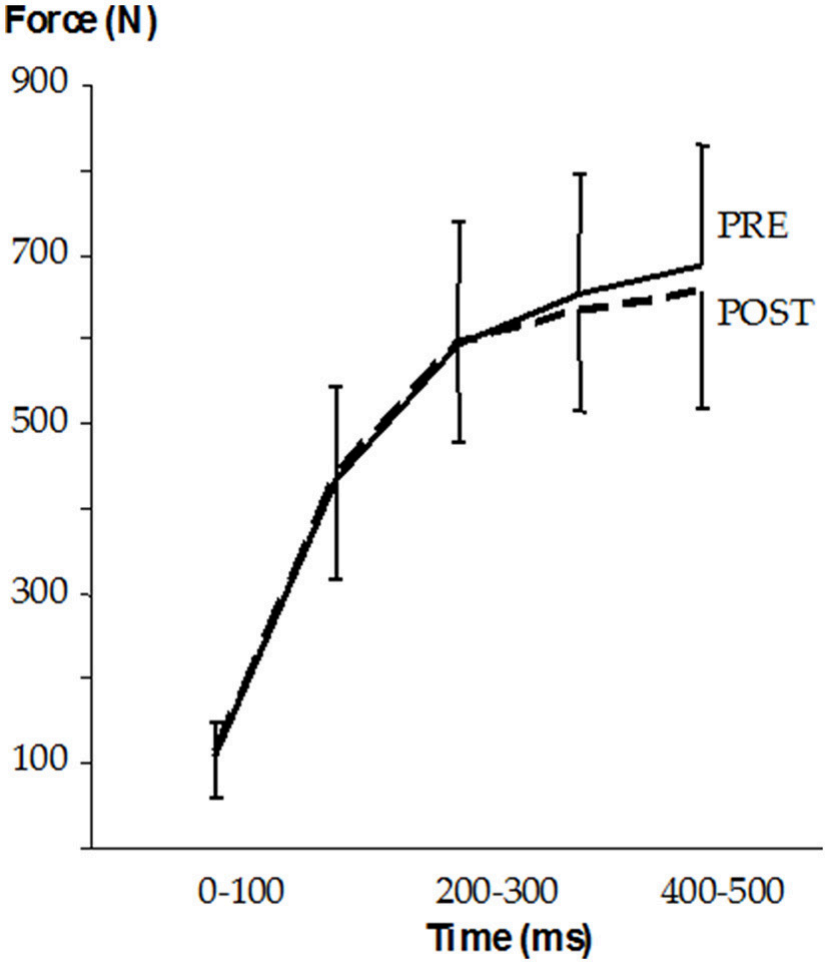
Mean (±SD) force-time curves before (PRE) and after (POST) the SD.

Maximal isometric force did not change after SD (PRE: 778 ± 166 vs. POST: 754 ± 162 N) and similarly, no changes were noticed in the rate of force production and force-time curves (Figure 5). The reflex amplitudes of the vastus lateralis muscle (PRE: 0.626 ± 0.441 vs. POST: 0.669 ± 0.371 mV) and the respective forces (PRE: 44 ± 25 vs. POST: 39 ± 19 N) were also statistically unchanged.

### Motor control and balance

*In the target movement protocol pairwise testing* revealed a significant tendency for improved accuracy after 24 h of SD in the 20° condition (*p* < 0.05), whereas no changes were observed for the 60° condition (Table 1). In addition, the angular velocity increased in the 60° condition after 36 h of SD (pairwise *p* < 0.05) but no changes were detected in the 20° condition (Table 1). The reaction times improved for light stimulus at 36 h and sound stimulus at 24 h (pairwise *p* < 0.05) and remain thereafter unchanged. Detailed statistical results regarding ANOVA main effects including degree of freedom, mean square and effects size (partial eta square) are presented in the Supplementary Table 3.

**Table 1:** The results (mean ± SD) (95% CI) in the target movement protocol and simple reaction time tests during the SD.

	**2 h**	**12 h**	**24 h**	**36 h**	**48 h**	**60 h**
**TARGET MOVEMENT TEST**						
Error of the displacement in 60° range of motion after elbow flexion (deg)	5.2 ± 1.8 (4.4–6.1)	4.6 ± 1.8 (3.7–5.4)	4.4 ± 1.7 (3.6–5.2)	4.5 ± 1.8 (3.7–5.3)	4.5 ± 2.1 (3.5–5.4)	5.3 ± 2.6 (4.1–6.5)
Error of the displacement in 60° range of motion after elbow extension (deg)	2.6 ± 1.3 (2.0–3.2)	2.7 ± 0.9 (2.2–3.1)	2.3 ± 1.0 (1.8–2.8)	2.3 ± 1.1 (1.8–2.8)	2.5 ± 0.9 (2.1–3.0)	2.3 ± 0.8 (2.0–2.7)
Error of the displacement in 20° range of motion after elbow flexion (deg)	7.7 ± 3.4 (6.1–9.3)	7.7 ± 4.0 (5.9–9.6)	6.5 ± 2.9 (5.2–7.9)#	6.5 ± 3.1 (5.1–8.0)	6.1 ± 3.5 (4.4–7.7)^*^#	6.0 ± 2.3 (5.0–7.1)^*^#
Error of the displacement in 20° range of motion after elbow extension (deg)	3.0 ± 1.6 (2.2–3.7)	2.7 ± 1.8 (1.8–3.5)	2.1 ± 1.2 (1.6–2.7)^*^	2.3 ± 1.4 (1.6–2.9)	2.0 ± 1.3 (1.4–2.6)^*^#	1.9 ± 1.0 (1.4–2.3)^*^#
Angular velocity in 60° range of motion (deg·s^−1^)	534 ± 80 (499–569)	577 ± 85 (540–614)	591 ± 68 (561–621)	614 ± 61 (587–641)^*^	613 ± 70 (582–644)^*^	628 ± 70 (597–659)^*^
Angular velocity in 20° range of motion (deg·s^−1^).	295 ± 58 (270–320)	326 ± 71 (295–327)	305 ± 46 (285–325)	319 ± 55 (295–343)	307 ± 64 (279–335)	315 ± 51 (293–337)
**SIMPLE REACTION TIME TESTS**						
Reaction time test for light stimulus (ms)	105 ± 16 (97–112)	94 ± 17 (86–102)	91 ± 16 (83–92)	85 ± 17 (77–93)^*^	86 ± 14 (80–93)^*^	83 ± 16 75–90)^*^
Reaction time test for sound stimulus (ms)	103 ± 18 (94–110)	93 ± 18 (84–101)	84 ± 20 (75–94)^*^	84 ± 17 (76–92)^*^	86 ± 16 (78–93)^*^	82 ± 14 (76–89)^*^

* *Significant difference compared to baseline (2 h). p < 0.05*;

# *significant difference compared to 12 h*.

In the balance test, medio-lateral distance traveled decreased in both eyes open and eyes closed condition (pairwise *p* < 0.05) after 24 h of SD and were maintained afterwards. Moreover, in eyes closed condition the medio-lateral distance and mean velocity were decreased in the beginning of the SD period (pairwise *p* < 0.05) but increased back to baseline level toward the end of SD. In the tandem condition with eyes open, the medio-lateral, and antero-posterior distances were increased after 48 h and with eyes closed, the medio-lateral and antero-posterior distance after 36 h and the antero-posterior distance after 48 h (pairwise *p* < 0.05; Table 2). Detailed statistical results regarding ANOVA main effects including degree of freedom, mean square and effects size (partial eta square) are presented in the Supplementary Table 4.

**Table 2:** The results (mean ± SD) (95% CI) in balance during the SD (EO, eyes open; EC, eyes closed).

	**2 h**	**12 h**	**24 h**	**36 h**	**48 h**	**60 h**
EO: Medio-lateral distance (mm)	107 ± 39 (89–125)	98 ± 25 (86–109)	84 ± 17 (77–92)^*^#	91 ± 22 (80–101)^*^	86 ± 22 (76–96)^*^#	85 ± 25 (73–96)^*^#
EO: Antero-posterior distance (mm)	141 ± 35 (124–156)	136 ± 28 (122–149)	143 ± 31 (128–156)	140 ± 28 (127–153)	138 ± 30 (124–152)	146 ± 41 (127–165)
EO: Moment of velocity (mm/s)	7 ± 5 (5–10)	6 ± 2 (5–7)	6 ± 2 (5–7)	6 ± 2 (5–6)	6 ± 3 (5–7)	6 ± 3 (4–7)
EC: Medio-lateral distance	115 ± 30 (101–129)	104 ± 25 (92–115)	98 ± 25 (86–109)^*^	97 ± 26 (84–109)^*^	100 ± 25 (88–112)^*^	97 ± 30 (82–111)^*^
EC: Antero-posterior distance	234 ± 81 (196–272)	205 ± 57 (178–231)^*^	211 ± 67 (179–242)	212 ± 71 (178–244)	237 ± 82 (199–275)#Δ	234 ± 88 (192–274)#
EC: Moment of velocity (mm/s)	10 ± 4 (8–12)	8 ± 3 (7–10)	9 ± 5 (6–11)	7 ± 4 (5.3–9.2)^*^□	11 ± 6 (8–14)#Δ	10 ± 7 (7–13)Δ
Tandem/ EO: Medio–lateral distance	293 ± 76 (257–328)	295 ± 74 (260–330)	292 ± 64 (262–321)	286 ± 69 (254–318)	321 ± 64 (291–351)□Δ	328 ± 89 (286–369)□Δ
Tandem/ EO: Antero–posterior	255 ± 81 (217–293)	247 ± 66 (216–278)	265 ± 97 (220–310)	247 ± 90 (204–288)	276 ± 94 (231–319)Δ	269 ± 92 (225–311)
Tandem/ EO: Moment of velocity (mm/s)	49 ± 24 (37–60)	47 ± 21 (38–57)	47 ± 22 (37–58)	48 ± 32 (33–63)	61 ± 36 (44–77)	60 ± 30 (46–74)
Tandem/EC: Medio-lateral distance	669 ± 269 (543–795)	613 ± 176 (530–694)	653 ± 198 (560–746)	681 ± 232 (572–789)#	726 ± 256 (607–846)#	681 ± 220 (578–784)#
Tandem/ EC: Antero-posterior	546 ± 284 (413–679)	482 ± 171 (402–562)	537 ± 246 (421–651)	530 ± 248 (413–646)	567 ± 269 (441–693)#	546 ± 274 (417–673)
Tandem/ EC: Moment of velocity (mm/s)	283 ± 469 (64–502)	154 ± 71 (121–187)	183 ± 110 (131–234)	174 ± 125 (115–231)	203 ± 134 (140–266)	190 ± 135 (126–252)

* *Significant difference compared to baseline (2 h). p < 0.05*;

# *significant difference compared to 12 h. p < 0.05*;

□ *significant difference compared to 24 h. P < 0.05*;

Δ *significant difference compared to 36 h. p < 0.05*.

## Discussion

We hypothetized that 60-h SD would lead to changes in aerobic performance, whereas neuromuscular performance would be maintained. Interestingly, the present results demonstrated that 60-h SD did not cause changes in maximal aerobic or maximal neuromuscular performances. Nevertheless, significantly decreased cardiorespiratory values were observed during submaximal aerobic performance after SD. Furthermore, as hypothetized motor control and balance were slightly affected, mainly after 36–48 h of SD, however, no consistent patterns of changes were observed.

Similar to the present study findings, an earlier study has shown time to exhaustion to be unchanged after 60-h SD (Goodman et al., 1989). However, some studies have shown reduced time (Martin, 1981; Martin and Chen, 1984; Bond et al., 1986; Chen, 1991; Azboy and Kaygisiz, 2009; Temesi et al., 2013). Furthermore, previous studies have observed contradictory findings in maximal VO_2_, ventilation and RER in regard to maximal aerobic performance after SD showing either decreases (Bond et al., 1986; Plyley et al., 1987; Chen, 1991) or no changes (Martin and Gaddis, 1981; Goodman et al., 1989). In addition, no change in maximal heart rate was observed in the present study, which is in line with some earlier studies (Plyley et al., 1987; Goodman et al., 1989) and contradictory to the other ones (Martin and Gaddis, 1981; Bond et al., 1986; Chen, 1991). The conflicting results between studies may depend on several issues, such as small sample size resulting in low statistical power, differences in experimental environment, exercise protocols used, fitness levels of the participants and even intra-individual responsiveness to SD (Fullagar et al., 2015).

Interestingly, SD affected cardiorespiratory responses in the submaximal level of performance resulting in decreased values of heart rate, oxygen consumption and ventilation after SD. These findings are in line with several previous studies (Martin and Haney, 1982; Bond et al., 1986; Azboy and Kaygisiz, 2009; Oliver et al., 2009), but not all (Martin, 1981; Martin and Gaddis, 1981; Martin and Chen, 1984; Plyley et al., 1987; Chen, 1991; Temesi et al., 2013). Currently, the biological mechanisms behind the changes in cardiorespiratory responses during exercise after SD are, however, poorly understood. Earlier findings with this same study sample showed, however, altered autonomic nervous system regulating cardiac function (heart rate variability), namely increased parasympathetic activity resulting as lower heart rate in resting condition (Vaara et al., 2009). The present findings together with the earlier findings may indicate down-regulatory responses from the cardiovascular regulation center. Interestingly, this physiological phenomenon seems to be similar what has been observed in athletes with functional overreaching. Aubry et al. (2015) observed that immediate heart rate recovery from maximal performance was in fact significantly faster in functionally overreaching triathletes compared to normal training state. The authors concluded that this could be induced by a decreased central command and by a lower chemoreflex activity. Speculatively, these two different stress sources, SD and arduous physical training, may result as a similar physiological changes, namely vagal hyperactivity, resulting as lower heart rate at rest and submaximal working intensity. It must be noted, however, that traditionally chronic training adaptations results in lower responses for a given work load in cardiorespiratory function, such as for example lower heart rate, which was observed after SD in the present study. In the absence of either decreased or increased time to exhaustion it remains controversial whether these altered responses to submaximal work is related to performance. In addition, changes in blood plasma volume and substrate oxidation could be factors linked to attenuated submaximal cardiorespiratory responses. However, RER values were not affected by the SD and therefore substrate utilization seemed not to be affected by SD. On the other hand, some decreases in LA concentration were observed. Previous studies have observed SD to reduce insulin sensitivity and glucose tolerance mechanisms (Knutson et al., 2007), which may in part be responsible for the decreased LA concentration in the present study through these mechanisms. The decreased LA at maximal level occurring after SD resembles to attenuated LA responses of athletes with functional overreaching (Aubry et al., 2015).

Interestingly, based on the present findings, the lower submaximal responses in cardiorespiratory variables were not evident regarding maximal performance level. It has previously been speculated that central or peripheral fatigue could explain the changes in aerobic performance but, however, a recent study did not confirm this (Temesi et al., 2013). Furthermore, Oliver et al. (2009) reported that SD did not induce changes in treadmill test during submaximal steady-state work (60% VO_2_max) but it decreased in the higher intensity distance test. This might indicate that the effect of SD may be a threshold-specific across the intensity of the performance or dependent on the length of the performance test used. It is also likely that the longer SD period, such as in the present study, may induce larger changes in submaximal performance. Moreover, some studies have observed that economic cost can be decreased by prolonged physical activity combined with SD, such as ultramarathon races (Vernillo et al., 2016, 2017). Although, these mechanism may be not applicable to the present study where participants were sedentary during SD, economic cost of movement may be one factor mediating physical performance outcomes in SD studies involving high physical activity.

In the present study, no changes either in maximal isometric strength, rate of force development or EMG of the knee extensors were observed after the 60-h SD. These findings are well in line with previous studies that assessed maximal force production after 24-h SD (Meney et al., 1998; Temesi et al., 2013), 48-h SD (Rodgers et al., 1995), and 60-h SD (Symons et al., 1988b). In contrast, one earlier study reported decrements in vertical jump and isokinetic force after 64-h SD, however, no changes in maximal isometric grip strength or isokinetic endurance test were observed (Takeuchi et al., 1985). Moreover, two studies, which combined SD and exercise found decreased maximal force production (Bulbulian et al., 1996; Skein et al., 2011) and decreased voluntary activation (Skein et al., 2011). However, it is difficult to interpret the independent effect of SD when these studies combined SD and exercise, especially when Skein et al. (2011) additionally reported reduced muscle glycogen concentration. Collectively, the current evidence of the effect of SD on physical performance indicates a greater influence on cardiorespiratory performance rather than muscular performance. However, there are only few studies that have assessed maximal neuromuscular performance and SD and, furthermore, there are no studies that have assessed repeated submaximal force production during SD. It has been suggested, based on partial SD studies, that submaximal prolonged tasks requiring strength capabilities may be more affected than short maximal tasks (Halson, 2014; Fullagar et al., 2015). In addition, controversial findings have been observed between isometric, isokinetic, and dynamic test procedures within single studies showing decreases in some measures of strength but no changes in others (Takeuchi et al., 1985; Bulbulian et al., 1996). Nevertheless, there are no consisting patterns whether dynamic and isometric strength is affected similarly or differently by SD.

SD did not induce severe decrements in balance, reaction times, or in motor control assessed by target movement measures in the present study. In fact, after 24 h some of the parameters showed improved values and thereafter they remained unchanged. However, the opposite was observed in the balance test with eyes closed. Previous studies have mostly shown SD to induce reductions in motor control ability (Fabbri et al., 2006; Forsman et al., 2007; Morad et al., 2007; Gomez et al., 2008; Bougard et al., 2011). Interestingly, a study by Patel et al. (2008) observed postural control decrements after 24 h of SD, whereas further decrements were not observed until 36 h of SD, being in line with the present study results. Similarly, many of the previous studies have reported reaction times to increase as a result of SD (Fullagar et al., 2015). Nevertheless, a study by Symons et al. (1988a) observed no detrimental changes in line with the present study. The possible explanations behind the plateau in motor control and reaction time decrements may include learning effect which could have been taken place in the beginning of SD (<24 h) and which would further mask the decrease in later phase regarding the present and a previous study (Patel et al., 2008). Furthermore, the participants of the present study were all well experienced with extended wakefulness (SD) due to their education as military officers, during which they have had several military field trainings with SD prior to the present study. Moreover, previously it has been observed that SD induces decrements in long and monotonous tasks to a higher extent compared to shorter ones (Pilcher and Huffcutt, 1996), which could partially explain the differences in observations in the present and a previous study (Patel et al., 2008).

Some limitations must be taken into account in the present study. The lack of a control group may be considered as a weakness of the present study. However, considering the unique nature and setting in this military training period during which the study was executed, we were not able to recruit a control group or to use a cross-over design. The strength of the current study was extensive measures on different dimensions of physical performance and motor control.

In conclusion, the present findings showed that both maximal neuromuscular and maximal aerobic performances are unaffected after 60-h SD, and similarly, motor control and simple reaction time can mostly be maintained. Therefore, it may be suggested based on present findings that in military or other physically demanding occupations the working demands regarding cardiorespiratory, neuromuscular, or motor control at maximal level can mostly be met even when exposed to SD. Nevertheless, as military work most often consists of submaximal physical activity intensities the present study findings that submaximal cardiorespiratory responses to SD was decreased, is an interesting finding. However, this finding together with previous findings that show alterations in glycogen metabolism and metabolic rate after SD reveal different physiological responses induced by SD. Whether these physiological alterations in submaximal level may ultimately have an deteriorative effect on physical performance when prolonged physical activity is maintained, such as in ultra endurance events and in military work, is an interesting topic for future research.

## Author contributions

JV, HO, HeK, MV, and TF conceived and designed the experiments. JV and HO performed experiments and analyzed data. JV, HO, HaK, MV, and TF interpreted results of research. JV, HO, HeK, MV, HaK, and TF drafted, edited, critically revised paper, and approved final version of manuscript.

### Conflict of interest statement

The authors declare that the research was conducted in the absence of any commercial or financial relationships that could be construed as a potential conflict of interest.
