# Supplementary material for: 60-Hour Sleep Deprivation Affects Submaximal but Not Maximal Physical Performance
Source: Front Physiol. 2018 Oct 16;9:1437. doi: 10.3389/fphys.2018.01437 (PMC6198717; doi:10.3389/fphys.2018.01437)
Supplement: Supplementary file 1 [file Table_1.pdf]

Supplementary table 1. Performance test results (mean±sd) (95%CI) during the bicycle ergometer test by each test bout. (All subjects were able to cycle at least 7 minutes, but only 4 subjects completed 10 minutes of cycling).

	**1 min** (n=10)	**2 min** (n=10)	**3 min** (n=10)	**4 min** (n=10)	**5 min** (n=10)	**6 min** (n=10)	**7 min** (n=10)	**8 min** (n=9)	**9 min** (n=8)	**10 min** (n=4)	**Maximal result**
**HR**: Pre (b/min)	125±14 (116-135)	134±15 (123-144)	142±17 (130-154)	150±17 (138-162)	158±16 (146-170)	167±15 (157-177)	174±13 (165-183)	174±12 (164-183)	183±10 (173-191)	185±10 (170-200)	190±7 (185-195)
**HR**: Post (b/min)	111±9 (104-118) **	122±11 (114-129) **	131±13 (122-140) **	138±13 (129-147) **	148±15 (138-159) **	156±13 (146±166) **	161±13 (151-170) *	169±12 (160-178)	173±10 (163-182)	178±10 (168-189)	187±6 (182-191)
**HR**: Pre-Post difference (b/min)	-14±11 (-22 - -6)	-11±8 (-11 - -17)	-11±8 (-17 - -6)	-12±8 (-17 - -6)	-10±10 (-17 - -3)	-11±10 (-18 - 4)	-13±13 (-22 - -4)	-5±12 (-14 - 4)	-10±11 (-19 - 1)	-10±18 (-55 - 35)	-3.1±5.3 (-6.9 - 0.7)
**VO_2**: Pre ml·kg^{-1}·min^{-1})	12.4±3.1 (10.2-14.6)	20.8±4.2 (17.8-23.9)	24.8±4.5 (23.5-30.1)	26.8±4.6 (23.5-30.1)	29.3±4.8 (25.9-32.8)	31.4±5.1 (27.7-35.0)	33.8±5.6 (29.7-37.8)	35.9±6.2 (31.1-40.6)	36.9±6.9 (31.2-42.7)	37.8±9.1 (23.4-52.26)	38.6±8.1 (32.8-44.3)
**VO_2**: Post ml·kg^{-1}·min^{-1})	11.4±2.9 (9.3-13.5)	18.3±3.2 (16.0-20.6) **	21.2±3.5 (18.7-23.7) **	24.0±3.7 (21.3-26.7) **	26.5±3.9 (23.7-29.3) **	29.0±4.5 (25.8-32.2) *	31.1±4.7 (27.8-34.4) *	33.4±5.5 (29.2-37.6) *	34.7±5.5 (30.0-39.3)	36.6±5.9 (27.1-46.0)	38.5±6.4 (33.9-43.1)

**VO_2**: Pre-Post difference ml·kg^{-1}·min^{-1})	-1.0±2.7 (-2.9 - 0.9)	-2.5±2.4 (-4.2 - -0.8)	-3.6±2.3 (-5.3 - -1.9)	-2.8±2.2 (-4.3 - -1.3)	-2.8±2.5 (-4.6 - -1.0)	-2.4±2.9 (-4.4 - -0.3)	-2.7±2.7 (-4.7 - -0.7)	-2.5±2.9 (-4.7 - -0.3)	-2.2±3.3 (-5.0 - 0.5)	-1.3±5.1 (-9.4 - 6.8)	-0.1±3.1 (-2.3 - 2.2)
**VE**: Pre (L/min)	27.2±3.4 (24.7-29.6)	37.6±4.6 (34.3-40.9)	49.0±6.1 (44.6-53.4)	56.3±7.9 (50.6-61.9)	64.7±8.0 (59.0-70.4)	76.0±10.0 (68.9-83.2)	88.6±13.3 (79.0-98.1)	108.7±21.0 (92.5-124.9)	132.8±30.9 (107.0-158.6)	132.4±24.9 (92.9-172.0)	149.6±33.5 (125.7-173.5)
**VE**: Post(L/min)	23.2±2.4 (21.5-24.9) *	33.2±3.1 (31.0-35.3) *	41.2±3.9 (38.4-43.9) **	49.0±5.6 (44.9-53.0)	57.1±8.0 (51.3-62.8) *	68.2±9.8 (61.2-75.2) *	76.9±13.8 (67.0-86.8) *	94.0±19.5 (78.9-109.0) *	114.2±26.4 (92.2-136.2)	119.2±19.1 (88.7-149.6)	153.1±30.2 (131.5-174.6)
**VE**: Pre-Post difference (L/min)	-4.0±4.0 (-6.9 - -1.1)	-4.5±5.6 (-8.5 - -0.5)	-7.9±7.3 (-13.1 - -2.6)	-7.3±8.6 (-13.5 - -1.1)	-7.6±9.7 (-14.5 - -0.7)	-7.8±9.7 (-14.7 - -0.9)	-11.7±13.5 (-21.3 - -2.0)	-14.7±20.1 (-30.2 - 0.7)	-18.6±23.0 (-37.9 - 0.6)	-13.3±32.1 (-64.3 - 37.8)	3.5±11.8 (-5.0 - 11.9)
**RER:** Pre	1.05±0.13 (0.96-1.15)	0.99±0.07 (0.94-1.04)	0.87±0.08 (0.81-0.93)	0.96±0.11 (0.88-1.04)	1.05±0.11 (0.97-1.13)	1.11±0.13 (1.02-1.20)	1.18±0.15 (1.07-1.29)	1.23±0.15 (1.11-1.34)	1.32±0.21 (1.17-1.47)	1.36±0.23 (1.19-1.52)	1.42±0.30 (1.20-1.62)
**RER:** Post	0.99±0.12 (0.90-1.08)	0.92±0.10 (0.85-0.99)	0.84±0.07 (0.79-0.89)	0.92±0.11 (0.84-1.00)	0.99±0.10 (0.92-1.07)	1.05±0.09 (0.98-1.11)	1.12±0.10 (1.05-1.19)	1.15±0.10 (1.07-1.21)	1.23±0.13 (1.13-1.32)	1.26±0.13 (1.17-1.35)	1.30±0.12 (1.21-1.38)

**RER:** Pre-Post difference	-0.06±0.17 (-0.18 - 0.06)	-0.07±0.13 (-0.16 - 0.03)	-0.03±0.12 (-0.12 – 0.05)	-0.04±0.18 (-0.17 – 0.09)	-0.05±0.14 (-0.16 – 0.05)	-0.06±0.13 (-0.15 – 0.03)	-0.06±0.15 (-0.17 – 0.04)	-0.08±0.13 (-0.17 – 0.01)	-0.09±0.16 (-0.20 – 0.02)	-0.10±0.16 (-0.21 – 0.02)	- 0.12±0.20 (-0.26 – 0.02)
**LA**: Pre (mmol/L)	-	2.8±2.0 (1.4-4.2)	-	3.3±1.4 (2.3-4.2)	-	5.5±2.2 (3.9-7.1)	-	9.4±3.2 (6.9-11.9)	-	10.0±3.1 (5.1-14.9)	10.7±2.3 (9.0-12.5)
**LA**: Post (mmol/L)	-	1.9±0.7 (1.4-2.4)	-	2.6±0.6 (2.1-3.0)	-	4.2±1.0 (3.5-5.0)	-	6.5±1.6 (5.3-7.7) *	-	9.0±4.0 (2.6-15.4)	8.2±2.6 (6.2-10.2) *
**LA**: Pre-Post difference (mmol/L)		-0.9±1.9 (-2.3 - 0.4)	-	-0.7±1.1 (-1.5 - 0.3)	-	-1.3±1.9 (-2.6 - 0.1)	-	-2.9±2.5 (-4.8 - -1.0)	-	-2.0±1.4 (-14.7 - 10.7)	-2.5±2.6 (-4.5 - -0.6)

HR=heart rate, VO_2=oxygen uptake, VE=ventilation, LA=lactate * $p<0.05$ significant difference compared to PRE-value, ** $p<0.005$ significant difference compared to PRE-value
