# Supplementary material for: 60-Hour Sleep Deprivation Affects Submaximal but Not Maximal Physical Performance
Source: Front Physiol. 2018 Oct 16;9:1437. doi: 10.3389/fphys.2018.01437 (PMC6198717; doi:10.3389/fphys.2018.01437)
Supplement: Supplementary file 2 [file Table_2.pdf]

Supplementary table 2. The detailed statistics (degree of freedom, mean square, F-value, significance and partial eta square) in aerobic performance test before and after the SD.

Variable	df	Mean Square	F	Significance	Partial eta squared
**HR (**b/min)	1.0	4929.3	18.1	0.002	0.669
**VO_2** (ml·kg^{-1}·min^{-1})	1.0	225.9	14.7	0.004	0.621
**VE** (L/min)	1.0	1835.7	8.6	0.016	0.49
**RER**	1.0	0.104	1.8	0.213	0.166
**LA** (mmol/L)	1.0	44.2	8.0	0.022	0.501
