# Supplementary material for: 60-Hour Sleep Deprivation Affects Submaximal but Not Maximal Physical Performance
Source: Front Physiol. 2018 Oct 16;9:1437. doi: 10.3389/fphys.2018.01437 (PMC6198717; doi:10.3389/fphys.2018.01437)
Supplement: Supplementary file 3 [file Table_3.pdf]

Supplementary table 3. The detailed statistics (degree of freedom, mean square, F-value, significance and partial eta square) in motor control and reaction times.

**Variable**	**df**	**Mean Square**	**F**	**Significance**	**Partial eta squared**
Error of the displacement in 60 ° range of motion after elbow flexion (deg)	5	3.416	1.3	0.246	0.067
Error of the displacement in 60 ° range of motion after elbow extension (deg)	5	0.457	0.7	0.587	0.038
Error of the displacement in 20 ° range of motion after elbow flexion (deg)	5	11.532	3.3	0.008	0.150
Error of the displacement in 20 ° range of motion after elbow extension (deg)	3.164	5.711	4.4	0.06	0.188
Angular velocity in 60 ° range of motion ($deg{\cdot}s^{-1}$)	3.074	37470.335	16.1	<0.001	0.459
Angular velocity in 20 ° range of motion ($deg{\cdot}s^{-1}$).	5	2355.273	1.9	0.094	0.093
Reaction time test for light stimulus (ms)	2.988	2179.7	10.1	<0.001	0.346
Reaction time test for sound stimulus (ms)	3.290	1819.0	10.8	<0.001	0.363
