# Supplementary material for: 60-Hour Sleep Deprivation Affects Submaximal but Not Maximal Physical Performance
Source: Front Physiol. 2018 Oct 16;9:1437. doi: 10.3389/fphys.2018.01437 (PMC6198717; doi:10.3389/fphys.2018.01437)
Supplement: Supplementary file 4 [file Table_4.pdf]

Supplementary table 4. The detailed statistics (degree of freedom, mean square, F-value, significance and partial eta square) in balance test during the SD (EO=eyes open, EC=eyes closed).

**Variable**	**df**	**Mean Square**	**F**	**Signicance**	**Partial eta squared**
**EO:** Medio-lateral distance (mm)	2.60	3116.8	5.3	0.004	0.220
**EO:** Antero-posterior distance (mm)	5	246.6	0.5	0.698	0.028
**EO:** Moment of velocity (mm/s)	2.473	18.8	1.3	0.278	0.065
**EC:** Medio-lateral distance (mm)	5	1070.0	3.0	0.014	0.137
**EC:** Antero-posterior distance (mm)	5	4167.5	2.9	0.019	0.131
**EC:** Moment of velocity (mm/s)	3.184	54.2	2.8	0.045	0.128
**Tandem/ EO:** Medio-lateral distance	2.89	10274.1	2.5	0.067	0.118
**Tandem/ EO:** Antero-posterior	5	2875.5	0.8	0.542	0.041
**Tandem/ EO:** Moment of velocity (mm/s)	5	851.3	1.6	0.169	0.077
**Tandem/ EC:** Medio-lateral distance	5	27884.8	1.6	0.170	0.077

**Tandem/ EC:** Antero-posterior	3.293	24871.7	0.8	0.485	0.042
**Tandem/ EC:** Moment of velocity (mm/s)	1.273	159692.3	1.0	0.394	0.052
